# Prevention and management of enteral nutrition-related diarrhea in stroke patients: barriers and enablers from best evidence to best practice

**DOI:** 10.3389/fnut.2026.1816074

**Published:** 2026-05-19

**Authors:** Jing Qiu, Jie Qin, Ruo Zhuang, Yingchun Huan, Manling Li, Yuxue Tong, Hong Yin, Yan Liu, Wei Zong

**Affiliations:** 1 Department of Neurosurgery, Affiliated Hospital of Xuzhou Medical University, Xuzhou, China; 2 Department of Neurology, Affiliated Hospital of Jiangsu University, Zhenjiang, China; 3 Department of Operating Room, Yancheng First People's Hospital, Yancheng, China

**Keywords:** barriers, best evidence, diarrhea, enablers, enteral nutrition, Ottawa model of research use, stroke

## Abstract

**Purpose:**

This study aimed to identify the gap between the clinical practice and best evidence for preventing and managing enteral nutrition-related diarrhea in stroke patients, to recognize barriers and enablers in translating this evidence into best practice, and to develop specific and effective action strategies to advance evidence-based practice.

**Methods:**

A convergent mixed-methods study was conducted, integrating cross-sectional surveys with descriptive qualitative research. Twenty-nine audit criteria were developed based on the current best evidence for the prevention and management of enteral nutrition-related diarrhea in stroke patients. A self-designed audit sheet, which was based on the developed audit criteria, was administered to conduct a baseline audit to identify gaps between best evidence and current clinical practice. A self-designed questionnaire based on the current best evidence was used to collect data on healthcare professionals’ knowledge regarding the prevention and management of enteral nutrition-related diarrhea in stroke patients. In-depth interviews were used to produce in-depth views of stakeholders about implementing the best evidence for preventing and managing enteral nutrition-related diarrhea in stroke patients. The qualitative data was analyzed using directed content analysis, guided by the Ottawa Model of Research Use (OMRU).

**Results:**

A total of 86 stroke patients, 34 nurses, and 4 doctors were recorded for their adherence to best evidence. The audit results showed that compliance rates for 19 out of 29 audit criteria were below 60.00%, and the incidence of enteral nutrition-related diarrhea among stroke patients was 43.02%. Thirty-eight healthcare professionals from the neurology department completed the questionnaire. The average knowledge score of the prevention and management of enteral nutrition-related diarrhea among these professionals was 53.68 (SD 15.74) points, with a pass rate of 42.11%. A total of 8 healthcare providers and 8 stroke patients were interviewed. We generated 11 themes and 23 sub-themes based on the data, which were categorized under three aspects of OMRU. At the evidence-based innovation level, the evidence meeting clinical needs served as a facilitator, while the lack of specific implementation standards for some evidence acted as a barrier. At the level of potential adopters, healthcare professionals’ positive attitudes toward evidence implementation and their exceptional work capabilities served as enablers for the best practice in preventing and managing diarrhea related to enteral nutrition in stroke patients. However, a lack of initiative among medical staff and the increased workload from evidence implementation were barriers. At the practice environment level, comprehensive supporting facilities, positive interpersonal relationships, patients’ positive rehabilitation mindset, and their absolute trust in medical professionals were important enablers. Important barriers included non-establishment of a multidisciplinary team, insufficient organizational support (e.g., lack of diarrhea prevention and management systems, lack of a quality assessment system for enteral nutrition), patients’ lack of relevant knowledge, low self-efficacy, and patients’ diminished abdominal sensitivity. Eleven strategies were developed to amplify enablers and overcome barriers to the implementation of evidence-based practice in preventing and managing enteral nutrition-related diarrhea in stroke patients.

**Conclusion:**

This study systematically investigated the barriers and enablers contributing to the gaps between the best evidence and clinical practice regarding the prevention and management of enteral nutrition-related diarrhea in stroke patients. Action strategies to promote the implementation of best evidence should focus on addressing barriers related to evidence-based innovation, potential adopters, and the practice environment.

## 1 Introduction

Stroke is defined as a brain dysfunction caused by acute focal damage resulting from altered cerebral blood supply (1). Malnutrition is a common complication of stroke, closely associated with poor prognosis, slow functional recovery, and prolonged hospital stays (2, 3). Previous studies have demonstrated that adequate nutritional support is a key factor in improving patient recovery outcomes, reducing the risk of complications, mitigating neurological deficits, and lowering healthcare expenditures (3, 4). Enteral nutrition (EN) is generally preferred to parenteral nutrition because of its relative safety, simplicity, and lower cost, as well as its ability to prevent intestinal bacterial translocation and maintain mucosal barrier function (5, 6). Although early EN has been shown to improve neurological and cognitive function in stroke patients while reducing mortality and the prevalence of malnutrition, patients often experience early intolerance to EN (3, 5, 7). Studies indicated that the incidence of diarrhea during EN in stroke patients reaches as high as 27.7–38% (8, 9). Electrolyte imbalances, pressure ulcers, and secondary infections caused by diarrhea not only easily lead to or exacerbate malnutrition but also compromise patient dignity and diminish quality of life (10, 11). Hypovolemia resulting from severe diarrhea further intensifies the severity of ischemia in stroke patients, increasing mortality (12). Additionally, the need for recurrent perineal cleansing or changing soiled bedding linens due to diarrhea significantly increases the workload for caregivers (10). More notably, diarrhea may lead to the discontinuation of EN, resulting in inadequate energy and protein intake and subsequently adverse clinical outcomes (3, 13, 14).

Obviously, proactive prevention and scientific management of enteral nutrition-related diarrhea in stroke patients are crucial for improving patient health outcomes and reducing the burden of care. Evidence-based practice is a vital bridge between the best evidence and clinical practice. It has revolutionized traditional experience-based nursing models by emphasizing the application of best evidence in practice, and it is a key pathway for advancing the professionalization and scientific transformation of clinical practice.

However, currently evidence-based studies have primarily focused on the prevention and management of enteral nutrition-related diarrhea in adult inpatients or critically ill patients (15, 16). Due to significant differences in management priorities and content among various specialties, their guidance on the systematic management of enteral nutrition-induced diarrhea in stroke patients is rather limited. Therefore, there is a need to develop evidence-based practices tailored to this unique population. What’s more, because healthcare facilities at different levels vary in terms of human resources, organizational environments, management mechanisms, and feeding strategies, there’s still a big gap between actual clinical practice and the best available evidence (9, 17, 18). For example, only 43.5% of hospital stroke units have established multidisciplinary teams, and only 22% of nurses reported that their departments have established protocols or systems related to EN (19). The guidelines state that continuous nasal feeding, rather than intermittent nasal feeding, should be used for patients with severe stroke and those in the acute phase of stroke (18, 20). However, in actual clinical practice, only 54.6% of stroke patients currently use enteral feeding pumps (19). Majid and his team found clear differences in how nurses and dietitians handle patients with diarrhea. For instance, 97% of dietitians would adjust the EN formula, but only 19% of nurses would take this step. On the other hand, 54% of nurses stuck to checking hygiene rules regularly, while just 6% of dietitians did the same (21). These gaps make it harder to reduce how long patients have diarrhea and to make clinical care more standardized.

Furthermore, previous studies have largely focused on the prevention and management of a single aspect, such as the identification of risk factors or medication adjustments (8, 10), the selection of appropriate nutritional formulas or feeding routes (20, 22), attention to catheter maintenance (23), infusion methods (17), or infusion temperature (24), as well as the combined use of probiotics (25). However, there has been a striking lack of focus on the barriers that stand in the way of, and the enablers that could facilitate, the clinical translation of the best evidence for preventing and managing enteral nutrition-related diarrhea in stroke patients. More importantly, this area is precisely the key variable determining whether the best evidence can be successfully translated into best practice. Thus, to develop targeted strategies, promote the translation of evidence into clinical practice, and standardize the prevention and management of enteral nutrition-related diarrhea in stroke patients, it is necessary to systematically investigate the barriers and enablers contributing to the gaps between the best evidence and clinical practice.

Therefore, this study aimed to develop audit criteria that reflected clinical effectiveness and appropriateness based on the best evidence regarding the prevention and management of enteral nutrition-related diarrhea in stroke patients. Then, the practices of healthcare professionals were compared with the audit criteria to identify the specific gaps between best evidence and clinical practice. Meanwhile, guided by the Ottawa Model of Research Use (OMRU), stakeholders were interviewed in depth, and the barriers and enablers contributing to various gaps between the best evidence and clinical practice were systematically analyzed. Based on this, areas in need of improvement were identified, and targeted strategies were developed to promote evidence-based practice, reduce the incidence of enteral nutrition-related diarrhea in stroke patients, improve their nutritional status, and facilitate recovery.

## 2 Methods

### 2.1 Study design and theory

This study was conducted at the Affiliated Hospital of Jiangsu University, whose department of neurology is a nationally recognized key specialty with medical expertise ranking among the top in China. A convergent mixed-methods study was conducted, integrating cross-sectional surveys with descriptive qualitative research. Data was collected using structured observation, self-reporting, and face-to-face semi-structured interviews. First, twenty-nine audit criteria were developed based on the current best evidence for the prevention and management of enteral nutrition-related diarrhea in stroke patients (26). A self-designed audit sheet, which was based on the developed audit criteria, was administered to conduct a baseline audit to evaluate compliance with best practices. Second, a self-designed questionnaire based on current best evidence was used to collect data on healthcare professionals’ knowledge regarding the prevention and management of enteral nutrition-related diarrhea in stroke patients. Third, in-depth interviews were employed to get views regarding the prevention and management of enteral nutrition-related diarrhea from medical staff, leaders, and stroke patients. We used the OMRU to develop an interview outline and review and structure the collected data (27). This model aims to provide an interdisciplinary, comprehensive, and practical research framework. It can be applied across all levels of healthcare to support researchers in carrying out evidence-based practice. The OMRU emphasizes that six key factors serve as the core components in the knowledge translation process, namely evidence-based innovation, potential adopters, practice environment, intervention strategies, adoption of change, and outcome evaluation. This model emphasizes analyzing barriers and facilitators across three critical domains: evidence-based innovation, potential adopters, and the practice environment. Evidence-based innovation includes two levels: development process and attributes of innovation. Potential adopters include five aspects: awareness, attitudes, knowledge/skills, concerns, and current practice. The practice environment includes five dimensions: patient, culture/society, structure, economic, and uncontrollable events. We primarily employed the COREQ (Consolidated criteria for reporting qualitative research) Checklist to report this study (28).

### 2.2 Cross-sectional survey

#### 2.2.1 Study steps and participants

First, before beginning this study, we summarized the best evidence for the prevention and management of enteral nutrition-related diarrhea in stroke patients, followed by using the Australian JBI Evidence Recommendation Grading System (2014 version) to evaluate the grade of evidence for the recommendations (29). As a result of the evidence synthesis, there were 28 pieces of evidence summarized across five key areas: organizational management, screening for risk factors of diarrhea, multi-dimensional prevention strategies, assessment and management of diarrhea, and dynamic monitoring (26).

Second, an expert panel was established comprising 10 members: one neurologist, one neurosurgeon, one nutritionist, one rehabilitation physician, two neurology specialty nurses, two neurosurgery specialty nurses, and two evidence-based nursing experts. All experts have over 10 years of experience in their respective fields. Next, the expert panel conducted a comprehensive review of the evidence’s feasibility, appropriateness, meaningfulness, and effectiveness (FAME) (30). Based on the results of the FAME assessment, a series of expert panel discussions were subsequently conducted. These discussions aimed to develop precise, clearly defined, and quantifiable audit criteria while clarifying the subjects of clinical audits, outlining audit methodologies, and standardizing audit processes (15). Ultimately, twenty-nine audit criteria were developed based on the current best evidence for the prevention and management of enteral nutrition-related diarrhea in stroke patients (26). A self-designed audit sheet, which was based on the developed audit criteria, was administered to facilitate data collection. Then, the practices of healthcare professionals are compared with the audit criteria to identify the specific gaps between best evidence and clinical practice. The audit sheet consisted of two sections: section one collected patient basic information (including age, gender, diagnosis, NIHSS score, etc.); section two comprised 29 audit criteria covering five aspects: organizational management, screening for risk factors of diarrhea, multi-dimensional prevention strategies, assessment and management of diarrhea, and dynamic monitoring (see Supplementary File S1).

Third, based on the current best evidence for preventing and managing enteral nutrition-related diarrhea in stroke patients (26), a self-designed knowledge questionnaire was developed to assess the knowledge level of 38 medical staff members in the neurology department regarding diarrhea prevention and management (see Supplementary File S2). The first section of the questionnaire collected basic information from medical personnel, including gender, professional title, age, educational background, and years of service. The second section comprised 15 single-choice questions, 5 true/false questions, and 5 multiple-choice questions. Correct answers earn points, while incorrect answers do not. The total score is out of 100 points. This questionnaire covered multiple aspects, including the definition of enteral nutrition-related diarrhea, risk factors for enteral nutrition-related diarrhea in stroke patients, and the prevention and management of diarrhea. A score of ≥ 60 on the diarrhea knowledge questionnaire was considered passing. Difficulty is denoted by P, calculated as the average score divided by the full score; Discrimination (D) = P1 - P2 (where P1 = Difficulty for the top 27% high-scoring group, P2 = Difficulty for the bottom 27% low-scoring group) (31). The questionnaire in this study has a difficulty value of 0.54 and a discrimination value of 0.39, indicating moderate difficulty and good discrimination.

We conducted a baseline audit at the Affiliated Hospital of Jiangsu University from July to September 2025. The subjects of this survey included medical staff from the department of neurology and stroke patients with enteral nutrition. Inclusion criteria for medical personnel: (1) at least one year of work experience; (2) willingness to participate in this study. Exclusion criteria: (1) interns or trainees; (2) individuals on sick leave, personal leave, or maternity leave who have been continuously absent from their workstation for more than one month. Patient inclusion criteria: (1) patients diagnosed with stroke via CT or MRI according to the diagnostic criteria revised at the Fourth National Conference on Cerebrovascular Diseases (17); (2) aged 18 years old and above; (3) dysphagia and water swallow test ≥ 3 grade; (4) EN was initiated within 48 h of admission; (5) voluntary participation in this study. Exclusion Criteria: patients unable to cooperate with the study for any reason or who refuse to participate. Dropout Criteria: patients transferred to another department or who withdrew during the study, rendering further data collection impossible.

#### 2.2.2 Sample size

The sample size was calculated using the CreateMed online sample size calculator developed by Beijing Tianchuang Mingda Medical Technology Co., Ltd., with the formula for comparing two independent sample rates:

$$\mathrm{NA}=\mathrm{NB}=(\frac{P_{A}(1-P_{A})}{k}+P_{B}(1-P_{B})){\left(\frac{Z_{1-\alpha /2}+Z_{1-\beta }}{P_{A}-P_{B}}\right)}^{2}$$

According to previous research (32), the incidence of diarrhea in the control group was 35.71%, while that in the experimental group was 15.71%. With a test power of 1-*β* set at 0.80, a Type I error (*α*) of 5%, a two-tailed test, and a 1:1 sample ratio between groups, the calculated minimum sample size for each group is 72 cases. Accounting for a 10% loss to follow-up rate, the sample size for each group must be at least 80 cases. Ultimately, a total of 86 stroke patients receiving EN participated in the baseline audit.

#### 2.2.3 Data collection

An audit team was established, including two master’s degree students and three neurology nurses. The audit team members received standardized training prior to starting the audit. The training covered the study objectives, research content, and important considerations. The audit was conducted systematically, strictly adhering to the audit methods and verifying each standard individually. If the subject completed the item of audit criterion, mark “Yes” in the space; if not, mark “No.” The final compliance rate was calculated as (Number of compliant cases / Total cases investigated) × 100%. Participants were enrolled based on predefined inclusion/exclusion criteria, and the study’s purpose and significance were clearly communicated to them. The knowledge questionnaire employed a self-reporting method among healthcare personnel for data collection. At an appropriate time, the questionnaire was distributed to healthcare workers, who completed it independently. After 30 min, the questionnaires were collected on-site. Researchers cross-checked the returned questionnaires to verify the completeness of responses, thereby ensuring data validity.

#### 2.2.4 Data analysis

EpiData 3.1 was used for data entry and management, and the data entry quality was checked by the double data entry verification method. Statistical analysis was performed using SPSS 25.0 software. Shapiro–Wilk was employed to test the normality of data. The normal quantitative data was statistically described as mean and standard deviation (SD). The quantitative data with abnormal distribution was described as median and interquartile range (IQR). The qualitative data was statistically described as frequency (*n*) and percentage (%).

### 2.3 Descriptive qualitative research

#### 2.3.1 Study setting and participants

Based on the results of the baseline audit, stakeholders regarding audit criteria with a compliance rate below 60% were interviewed. Stakeholders include healthcare professionals and stroke patients. To gain a deeper understanding of stakeholders’ views in relation to the various levels (evidence content, individuals, organizations, and practice settings) that might have hindered or facilitated the implementation of best evidence for preventing and managing enteral nutrition-related diarrhea in stroke patients, we employed purposive sampling to recruit interview participants. Interviews commenced in October 2025 and were completed in November of the same year.

Inclusion criteria for medical personnel: (1) work in neurology medical practice, neurology nursing, or nursing management; (2) over 10 years of clinical experience; (3) educational qualification of bachelor’s degree or higher; (4) voluntary participation in this study. Exclusion criteria: (1) interns or trainees; (2) individuals on sick leave, personal leave, or maternity leave with continuous absence from work > 1 month.

Patient inclusion criteria: (1) patients diagnosed with stroke via CT or MRI according to the diagnostic criteria revised at the Fourth National Conference on Cerebrovascular Diseases (17); (2) aged 18 years old and above; (3) dysphagia and water swallow test ≥ 3 grade; (4) EN was initiated within 48 h of admission; (5) patients without impaired consciousness and with normal speech function; (6) voluntary participation in this study. Exclusion criteria: patients unable to cooperate with the study or refusing participation for any reason.

#### 2.3.2 Sample size

This qualitative study emphasized the richness of data rather than the number of informants providing information. Therefore, the sample size was determined by data saturation (33). When data saturation was reached (i.e., no new themes emerged), we continued to include three interviewees to ensure no information was omitted.

#### 2.3.3 Data collection

Following approval from hospital administration, WZ conducted data collection for the study. WZ holds a master’s degree in clinical nursing, possesses extensive research experience, and has received specialized training in both qualitative and quantitative research methodologies. Additionally, he is skilled at encouraging participants to speak freely. Based on the OMRU, we developed a preliminary semi-structured interview outline, which was subsequently revised by a nursing expert (RZ) and an expert in evidence-based methodology (YCH). The researcher WZ conducted pre-interviews with two healthcare professionals and two stroke patients using a revised outline to validate whether the interview questions could comprehensively and accurately gather relevant information. A final interview outline was constructed through step-by-step revision (see Supplementary File S3). Then, the interviewer selected eligible medical staff and patients in the neurology ward and confirmed the interview time and location with the participants. To minimize external interference, the setting for the interview was a quiet and private meeting room. Prior to conducting and audiotaping interviews, consent was obtained from the interviewee. Audio recordings were made with the interviewer’s smartphone to ensure documentation accuracy. The purpose, content, and significance of the study were first explained. Then, the basic information, such as the participant’s age, gender, marital status, and categories of interviewees (e.g., nurses, doctors, or patients), was recorded. Based on the interview outline, a semi-structured interview was conducted. Additionally, the researcher focused on interview content, facilitating participants’ articulation of authentic experiences while avoiding leading questions. At the same time, nonverbal expressions of the interviewees, including their emotions and body language, were systematically observed and documented. Each interview lasted between 30 and 60 min.

#### 2.3.4 Data management and analysis

Two researchers (WZ and JingQ) conducted data collection and analysis simultaneously. Within 48 h of each interview, researchers transcribed the audio recordings into written documents, then categorized and archived each file according to interview date, location, and category. NVivo 12.0 was used to manage data. Barriers and enablers were analyzed using OMRU theory across three dimensions: evidence-based innovation, potential adopters, and practice environment (27). The analyzing process follows the method recommended by Hsieh et al. (34) and Elo et al. (35). The specific steps are as follows: (1) Repeatedly read the textual materials to gain an overall impression; (2) Extract and code statements reflecting barriers or enablers; (3) Categorize the coded statements into corresponding dimensions of the OMRU framework; (4) Synthesize and refine the categorized content to generate themes and sub-themes; (5) Repeat the above steps until no new themes emerge. Two researchers reached consensus on the content of the data analysis through discussion. If disagreements arose during the analysis process, they consulted a third senior researcher (YL) to resolve the matter before proceeding to the next step. The findings are presented in themes and quotations, reflecting the barriers and enablers for stakeholders in applying the best evidence to prevent and manage enteral nutrition-related diarrhea in stroke patients.

### 2.4 Ethical consideration

This study was approved by the Ethics Committee of the Affiliated Hospital of Jiangsu University prior to implementation (No. KY2025H0717–05). Each participant signed an informed consent form. This study was conducted in accordance with the Declaration of Helsinki.

## 3 Results and findings

### 3.1 Participant characteristics

This baseline audit assessed adherence to best evidence among 86 stroke patients, with their general characteristics presented in Table 1. Thirty-eight healthcare professionals, including four neurologists and 34 neurology nurses, completed the knowledge questionnaire and underwent baseline assessments. Their ages ranged from 25 to 48 years, and their years of practice ranged from 3 to 25 years. Thirteen held junior professional titles, 22 held intermediate titles, and 3 held senior titles. A total of 16 stakeholders were interviewed, including 2 neurologists, 6 nurses, and 8 stroke patients. Their basic characteristics are shown in Table 2.

**Table 1:** General characteristics of stroke patients (*n* = 86).

Items	Characteristic	*n* (%)/M (SD)/median (IQR)
Gender	Male	59 (68.60)
	Female	27 (31.40)
Age	Years	67.23 ± 11.25
Marital status	Married	65 (75.58)
	Unmarried/divorced	21 (24.42)
Educational level	Junior high school and below	43 (50.00)
	Senior high	28 (32.56)
	Junior college and above	15 (17.44)
Medical payment mode	Health insurance/Commercial insurance	72 (83.72)
	Self-paying	14 (16.28)
Family monthly income (yuan)	<3,000	8 (9.30)
	3,000–8,000	68 (79.07)
	>8,000	10 (11.63)
Diagnose	Ischemic stroke	74 (86.05)
	Hemorrhagic stroke	12 (13.95)
Site of lesion	Left hemisphere	46 (53.49)
	Right hemisphere	40 (46.51)
NIHSS score		13.54 ± 3.85
GCS score		11.81 ± 1.58
Nasogastric feeding method	Nasogastric tube	80 (93.02)
	Nasojejunal tube	6 (6.98)
Along with other diseases	None	5 (5.81)
	Hypertension, diabetes, or coronary heart disease (any one of these conditions)	44 (51.16)
	Hypertension, diabetes, coronary heart disease (two of the three)	25 (29.07)
	Hypertension, diabetes, and coronary heart disease	7 (8.14)
	Diseases other than the three mentioned above	5 (5.81)
Length of Hospitalization	Day	10.87 ± 8.13

**Table 2:** Basic characteristics of stakeholders (*n* = 16).

Stakeholders group	*n*	%
Medical staff (*n* = 8)		
Neurologist	2	25.00
Nursing manager	2	25.00
Neurology nurse	4	50.00
Educational level		
Bachelor	5	62.50
Master	2	25.00
Doctor	1	12.50
Stroke patients (*n* = 8)		
Educational level		
Junior high school and below	3	37.50
Senior high	3	37.50
Junior college and above	2	25.00
Medical payment mode		
Health insurance/Commercial insurance	6	75.00
Self-paying	2	25.00

### 3.2 Baseline audit findings

Among the 86 stroke patients included in the study, 37 (43.02%) experienced diarrhea during EN. Compliance rates for 19 out of 29 audit criteria were below 60.00%, with 6 criteria showing zero adherence.

At the system level, the results for audit standards 1, 2, and 16 were all 0 indicating that the Department of Neurology had not yet established a multidisciplinary team, lacked a quality assessment system for EN, and did not have health education materials on the prevention and management of enteral nutrition-related diarrhea.

At the practitioner level, the audit criteria 3, 18, 20, 21, 22, 27, 28, and 29, which focused on nurses, yielded results of 0, 52.94, 5.88, 26.47, 0, 0, 5.88, and 47.06%, respectively. These results indicated that nurses demonstrated poor performance in actively screening stroke patients for risk factors of diarrhea; accurately applying diarrhea assessment tools; monitoring electrolyte levels, nutritional status, and mental status in patients with diarrhea; collecting blood and stool samples from patients with diarrhea for laboratory testing; reviewing medication lists for patients with diarrhea; and monitoring neurological function and biochemical laboratory parameters in patients with diarrhea. The audit criteria 7, 8, 10, 14, 15, 19, 23, and 25 were applied to healthcare professionals in the Department of Neurology, with all results showing 100%. These results indicated that healthcare professionals were highly competent in maintaining a clean operating environment, selecting appropriate EN formulas and routes, ensuring that the composition of nutritional preparations remains intact, keeping the nasogastric tube unobstructed, monitoring stool consistency and diarrhea frequency in patients with diarrhea, and slowing infusion rates and reducing total infusion volumes.

At the patient level, the audit criteria 4, 5, 6, 11, 12, 13, and 17, which focused on stroke patients, yielded results of 36.36, 37.33, 46.51, 67.44, 55.81, 67.44, and 17.44%, respectively. The results of audit standard 4 and audit standard 5 indicated that only 36.36% of patients took multiple measures to promptly correct hypoalbuminemia, while among patients with normal serum albumin levels, only 37.33% did not use protein supplements. The results of audit standard 6 indicated that only 46.51% of patients opted to use probiotics in conjunction with EN to prevent diarrhea. In other words, stroke patients possess limited knowledge about diarrhea prevention. Additionally, compliance with warming EN preparations for stroke patients in practice was only 55.81%. More notably, the implementation rate of providing EN education to stroke patients and assessing their knowledge retention through questioning was only 17.44%. The results of audit standard 11 indicated that the implementation rate of enteral feeding pumps among stroke patients was 67.44%. According to the results of audit standard 13, stroke patients had a 67.44% compliance rate with correct initial and maintenance infusion rates. The results of audit standard 9 indicated that the compliance rate for the proper storage of EN formulas for stroke patients was only 53.57%. In terms of diarrhea management, the results of audit standard 24 showed that only 10.81% of diarrhea patients consented to the changed EN formula. According to the results of audit standard 26, merely 27.02% of diarrhea patients were given perianal skin protectants. The specific audit criteria, audit subjects, audit methods, and audit results are presented in Table 3.

**Table 3:** Audit criteria, subjects, methods, and results for the prevention and management of enteral nutrition-related diarrhea in stroke patients.

Items	Audit criteria	Audit subjects	Audit methods	*n*	Y	Compliance rate (%)
Organizational management	1. The Department of Neurology has established a multidisciplinary team with clearly defined responsibilities to jointly manage the nutritional quality of stroke patients.	Department of Neurology	Consultation	1	0	0
	2. The Department of Neurology has established structure, process, and outcome assessment indicators related to enteral nutrition.	Department of Neurology	Review departmental files	1	0	0
Screening for risk factors of diarrhea	3. Neurological nurses perform a standardized risk assessment for diarrhea before administering enteral nutrition to stroke patients, evaluating three key aspects: the patient-related factors, the medication-related factors, and the enteral nutrition-related factors. (1) Patient-related factors: including NIHSS score, GCS score, advanced age, hyperglycemia, hypoalbuminemia, gastrointestinal infection, hemodynamic instability, fasting, Clostridioides difficile infection, lactate deficiency, or fat malabsorption. (2) Medication-related factors: including antibiotics, proton pump inhibitors, preparations containing sorbitol or electrolytes, acid suppressants, and stool softeners. (3) Enteral nutrition-related factors: including inappropriate formulations, improper feeding methods, unreasonable infusion concentration/rate/temperature, and microbial contamination.	Nurses	On-site observation Consultation	34	0	0
Multi-dimensional prevention strategies	4. Stroke patients with hypoalbuminemia take measures to correct hypoalbuminemia as directed by their physician.	Patients	On-site observation Consultation Review the medical orders	11	4	36.36
	5. Stroke patients with normal serum albumin levels do not require energy or protein supplementation.	Patients	On-site observation Consultation Review the medical orders	75	28	37.33
	6. Patients taking probiotics during enteral nutrition.	Patients	On-site observation Review the medical orders	86	40	46.51
	7. Nurses maintain the cleanliness of infusion lines and the operating environment during enteral feeding.	Nurses	On-site observation Consultation	34	34	100
	8. Neurologists select enteral nutrition formulas based on individual patient factors such as gastrointestinal function, comorbidities, and complications.	Neurologists	On-site observation Consultation Review the medical orders	4	4	100
	9. Once opened, the patient’s nutritional preparation is stored at room temperature for less than 4 h or at 4 °C for less than 24 h. Any unused portion after 24 h is discarded.	Nutritional preparations	On-site observation Consultation	28	15	53.57
	10. Neurologists select enteral feeding routes based on factors including the stroke patient’s nutritional risk, swallowing ability, level of consciousness, anticipated duration of enteral nutrition, and risk of complications.	Neurologists	On-site observation Consultation	4	4	100
Multi-dimensional prevention strategies	11. Neurology nurses selected enteral feeding pumps to provide continuous enteral nutrition infusion for patients. The disposable external feeding lines for enteral nutrition are replaced every 24 h.	Patients	On-site observation Consultation Review the medical orders Review the nursing records	86	58	67.44
	12. Nurses administer enteral nutrition preparations to patients at a temperature of 37 ~ 40 °C; for elderly patients, the temperature is maintained between 38 ~ 42 °C.	Patients	On-site observation Consultation Review the nursing records	86	48	55.81
	13. The patient’s enteral nutrition infusion rate on the first day is 20 ~ 50 mL/h; on the second day, the infusion rate is 80 ~ 100 mL/h; and within 3 ~ 7 days, it is increased to the full target volume.	Patients	On-site observation Consultation Review the nursing records	86	58	67.44
	14. The nurse did not add water or colored substances to the nutritional preparation.	Nurses	On-site observation Consultation	34	34	100
	15. During enteral feeding, nurses flush the feeding tube with 20 ~ 30 mL of warm water every four hours, and prior to and after interrupting feeding or administering medication.	Nurses	On-site observation Consultation Review the nursing records	34	34	100
	16. The neurology department has printed health education materials concerning the prevention and management of enteral nutrition-related diarrhoea in stroke patients.	Department of Neurology	On-site observation Review departmental files	1	0	0
	17. During enteral nutrition, nurses provide patients with education on enteral nutrition and assess their knowledge retention through questioning.	Patients	On-site observation Consultation Review the nursing records	86	15	17.44
Assessment and management of diarrhea	18. When a patient’s bowel movement frequency exceeds three times per day, nursing staff utilize the Diarrhea Scoring System to assess the patient’s diarrhea condition.	Nurses	On-site observation Consultation Review the nursing records	34	18	52.94
	19. When a patient presents with diarrhea, the nurse assesses abdominal signs, duration of diarrhea, frequency of bowel movements, and stool consistency.	Nurses	On-site observation Consultation Review the nursing records	34	34	100
	20. When a patient develops diarrhea, the nurse assesses the patient’s electrolyte levels, nutritional status, and mental state.	Nurses	On-site observation Consultation Review the nursing records	34	2	5.88
	21. When a patient experiences diarrhea, the nurse promptly collects blood and stool samples for laboratory testing.	Nurses	On-site observation Consultation Review the medical orders	34	9	26.47
Assessment and management of diarrhea	22. When a patient develops diarrhea, the nurse reviews all medications administered to the patient and collaborates with the doctor to make a joint decision regarding the suspension or replacement of the medication causing the diarrhea.	Nurses	On-site observation Consultation Review the medical orders	34	0	0
	23. When the patient experiences diarrhea, the nurse reduces the infusion rate of the enteral nutrition solution.	Nurses	On-site observation Review the nursing records	34	34	100
	24. When the patient experiences diarrhea, switch to a lactose-free or fiber-enriched formula.	Patients with diarrhea	On-site observation Review the medical orders Review the nursing records	37	4	10.81
	25. When a patient experiences diarrhea, the nurse reduces the total volume of enteral nutrition administered.	Nurses	On-site observation Consultation	34	34	100
	26. When a patient experiences diarrhea, the nurse observes the condition of the perianal skin and applies a skin protectant.	Patients with diarrhea	On-site observation Consultation Review the nursing records	37	10	27.02
Dynamic monitoring	27. When the patient experiences diarrhea, nurses employ the NIHSS score or GCS score to monitor neurological function in stroke patients.	Nurses	On-site observation Consultation Review the nursing records	34	0	0
	28. Nurses monitor stroke patients weekly for anthropometric measurements (body mass index, triceps skinfold thickness, muscle condition, etc) and biochemical laboratory parameters (complete blood count, electrolytes, total protein, albumin, prealbumin, etc.).	Nurses	On-site observation Consultation Review the nursing records	34	2	5.88
	29. Nurses assess and document changes in gastrointestinal function in stroke patients every 4 hours.	Nurses	On-site observation Review the nursing records	34	16	47.06

NIHSS, National Institutes of Health Stroke Scale; GCS, Glasgow Coma Scale; Y-The audit subjects performed correctly in completing the audit standard; Compliance rate = number of compliant cases (Y) / total cases investigated (*n*) × 100%.

### 3.3 Knowledge questionnaire results

Results indicated an average knowledge score of 53.68 (SD 15.74) points among medical staff, with a pass rate of 42.11% (16/38). The correct response rates for the appropriate temperature of enteral nutrition infusion, primary management measures for diarrhea, and the normal range of adult albumin were 36.84, 28.95, and 39.47%, respectively. Moreover, the correct response rates for multiple-choice questions remained low. This demonstrated inadequate mastery of diarrhea-related knowledge among healthcare professionals, necessitating enhanced training in this area. The results of healthcare personnel responses to the knowledge questionnaire are shown in Table 4.

**Table 4:** The results of healthcare personnel responses to the knowledge questionnaire (*n* = 38).

Items	Number of people who answered correctly, *n*	%
1. Definition of enteral nutrition-related diarrhea?	24	63.16
2. When do the guidelines recommend initiating enteral nutrition in stroke patients?	29	76.32
3. The appropriate infusion temperature for nutritional preparations is?	14	36.84
4. The correct initial infusion rate for enteral nutrition on the first day is?	28	73.68
5. The core principle for preventing enteral nutrition-related diarrhea is?​	28	73.68
6. What are the correct storage conditions for nutritional preparations?	27	71.05
7. Which disease is a risk factor for diarrhea?	31	81.58
8. Which types of medication can cause diarrhea?	18	47.37
9. Which component in nutritional preparations is most likely to cause osmotic diarrhea?	26	68.42
10. When patients experience abdominal pain and watery stools during enteral nutrition, the primary measure is?	11	28.95
11. What are the key indicators for assessing the degree of dehydration in patients with diarrhea?	36	94.74
12. Regarding the relationship between enteral nutritional diarrhea and antibiotics, the correct statement is?	20	52.63
13. For patients with enteral nutrition-related diarrhea, which aspects should be prioritized for monitoring?	27	71.05
14. The normal range for albumin in adults is?	15	39.47
15. Which intervention is ineffective for enteral nutrition-related diarrhea?	25	65.79
16. Patients with diarrhea should immediately take anti-diarrheal medication?	25	65.79
17. The severity of stroke patients’ condition is unrelated to diarrhea?	36	94.73
18. Serum prealbumin is a reliable indicator for assessing long-term nutritional status?	8	21.05
19. Routine supplementation with energy or protein preparations does not increase the risk of diarrhea in stroke patients with normal albumin levels?	17	44.74
20. Water or medication may be added to nutritional preparations?	38	100
21. What are the common causes of enteral nutrition-related diarrhea?	19	50.00
22. Key measures for preventing enteral nutrition-related diarrhea?	11	28.95
23. Which nutritional preparation is suitable for patients with indigestion?	4	10.53
24. What measures can be taken to improve enteral nutrition-related diarrhea?	7	18.42
25. What are the nutritional monitoring indicators for stroke patients?	15	39.47
Pass rate (number of successful candidates / total number of candidates)	16	42.11
Average score (point, mean ± SD)	53.68 ± 15.74	

### 3.4 Qualitative research findings

Based on the findings from 16 semi-structured interviews, we summarized the qualitative research results into 11 themes and 23 sub-themes using the OMRU framework. The identified barriers and enablers are presented in Table 5.

**Table 5:** Identified barriers and enablers.

**Level of OMRU**	**Theme**	**Sub-theme**	**Category**
Evidence-based innovation	Meeting clinical needs	The evidence aligns with national policy objectives	Enablers
		Addressing existing clinical issues	Enablers
		Resolving patients’ concerns	Enablers
	Some evidence lacks specific implementation standards	/	Barriers
Potential adopters	Lack of initiative	Insufficient awareness of diarrhea risk prevention	Barriers
		Knowledge gaps existence	Barriers
	Evidence implementations increase workload	/	Barriers
	Positive attitudes toward evidence implementation	/	Enablers
	Exceptional work capabilities	Strong execution capabilities	Enablers
		Strong sense of responsibility	Enablers
		High adapting ability	Enablers
		Extensive experience in evidence-based practice	Enablers
Practice environment	Comprehensive supporting facilities	/	Enablers
	Non-establishment of a multidisciplinary team	/	Barriers
	Insufficient organizational support	Lack of diarrhea prevention and management systems	Barriers
		Lack of a quality assessment system for enteral nutrition	Barriers
		Limited educational content and formats for diarrhea	Barriers
	Interpersonal relationships	Medical staff collaborate harmoniously	Enablers
		Support from leader	Enablers
		Collaboration-oriented interpersonal interaction	Enablers
	Patients	Lack of relevant knowledge	Barriers
		Low self-efficacy	Barriers
		Diminished abdominal sensitivity	Barriers
		Cognitive differences	Enablers/barriers
		‌Financial burden	Barriers
		Knowledge sharing among fellow patients	Enablers
		Positive rehabilitation mindset	Enablers
		Absolute trust in medical professionals	Enablers

#### 3.4.1 First level—evidence-based innovation

In this study, facilitators refer to elements mentioned in the feedback that promote evidence implementation, while barriers refer to any elements mentioned in the feedback that impede evidence application. At the level of evidence-based innovation, meeting clinical needs (i.e., the evidence aligns with national policy objectives, addresses existing clinical issues, resolves patient concerns) served as a facilitating factor, while the lack of specific implementation standards for some evidence constituted a barrier.

##### 3.4.1.1 Meeting clinical needs

- The evidence aligns with national policy objectives—meaning the evidence itself constitutes objective, verifiable information (e.g., nutritional status in stroke patients, strategies for preventing enteral nutrition-related diarrhea, neurological function scale scores), whose content or conclusions directly or indirectly point toward the goals pursued by national policy. For example, medical personnel said:

“Last year, the government launched a program to enhance nutritional management for stroke patients, emphasizing the prevention of avoidable complications arising from inadequate care […]. While neurological damage in stroke patients is irreversible, nutritional issues can be fully addressed through targeted interventions.” (Medical staff #1).

- Addressing existing clinical issues—refers to specific challenges identified by medical personnel during diagnosis, treatment, or nursing that require resolution. Medical personnel said:

“The incidence of diarrhea among stroke patients in our department is relatively high, posing a persistent challenge. The root cause has never been systematically investigated. I believe this evidence can effectively address the issue.” (Medical staff #2).

- Resolving patients’ concerns—diarrhea poses multiple challenges for stroke patients, including compromised dignity, disrupted social activities, and diminished quality of life. Healthcare providers proactively offering knowledge on diarrhea prevention and management can alleviate these various concerns for stroke patients. The patient said:

“Due to this condition, the right side of my body isn’t very agile. Sometimes I can’t make it to the bathroom in time, soiling my clothes and bedding, which makes me feel terribly embarrassed. I’m not quite sure why the diarrhea happens; I might think it’s because I caught a chill. It’s too difficult for me to ask the nurse directly about the specific cause. If you could proactively tell me how to prevent it, I’d be very grateful. It would resolve my confusion and protect my dignity.” (Stroke patient #1).

##### 3.4.1.2 Some evidence lacks specific implementation standards

For example, medical staff reported that identifying diarrhea risk in stroke patients involved numerous assessment items, and the absence of specific implementation standards led to variations in evaluation outcomes. This has become a primary concern for medical staff. Medical personnel said:

“There are so many risk factors for diarrhea in stroke patients […]. Yet there is no established standard for how to assess them, when to assess them, or what constitutes a comprehensive assessment. I believe this lack of standardization compromises the accuracy of assessment results.” (Medical staff #4).

#### 3.4.2 Second level—potential adopters

##### 3.4.2.1 Lack of initiative

- Insufficient awareness of diarrhea risk prevention—refers to the lack of comprehensive, scientific understanding of diarrhea risk factors and preventive measures, resulting in healthcare personnel failing to proactively prevent diarrhea during routine care. For instance, medical personnel said:

“The condition of stroke patients can change rapidly. My top priority is ensuring patient safety, so I focus primarily on changes in consciousness and limb movement, rarely paying attention to nutritional status. To be honest, I’m not very familiar with the prevention or risks associated with enteral nutrition-related diarrhea.” (Medical staff #7).

- Knowledge gaps exist—in this study, knowledge gaps refer to the cognitive lacunae among different stakeholders regarding the etiology and pathogenesis, risk identification, risk control, timing of intervention, and individualized management strategies for enteral nutrition-associated diarrhea in stroke patients. These gaps arise from inadequate professional training, inconsistent implementation of guidelines, or limited experience. Consequently, clinical decision-making deviates from evidence-based standards, thereby increasing the risk of diarrhea-related complications. They said:

“I always thought protein powder supplements were beneficial for stroke patients’ recovery. I never imagined that for patients with normal serum albumin levels, these supplements could increase the risk of diarrhea. I truly had no idea about this before.” (Medical staff #7).

“Since suffering a cerebral hemorrhage, my physical strength and energy have significantly declined compared to before. Coupled with my advancing age, my immunity has also weakened. Therefore, I have my spouse purchase various nutritional supplements for me to enhance my body’s resistance. I believe that taking more supplements will help my body recover faster.” (Stroke patient #6).

##### 3.4.2.2 Evidence implementations increase workload

Implementing best practice for preventing and managing enteral nutrition-related diarrhea in stroke patients increases the workload. On the one hand, diarrhea may be caused by multiple factors, including inappropriate nutritional solution formulation, excessive infusion rate, low temperature, or intestinal dysfunction or infection in the patient. Each of these factors must be systematically investigated. On the other hand, systematic diarrhea risk screening and variations in individualized care needs—such as patients’ bowel function, nutritional requirements, and skin condition—add complexity to nursing tasks. Medical personnel said:

“There are numerous daily nursing tasks to complete, and adding responsibilities like assessing diarrhea, monitoring nutrition, and providing health education not only increases the workload but also takes up a portion of my time. When things are less hectic, I’m willing to handle it, but when it gets busy, I probably won’t even remember.” (Medical staff #5).

##### 3.4.2.3 Positive attitudes toward evidence implementation

The positive attitude of stakeholders toward best practice for preventing and managing enteral nutrition-related diarrhea in stroke patients is a multidimensional clinical psychological concept. The core of this is the intrinsic motivation of stakeholders to proactively adopt best evidence across three dimensions: cognitive identification, affective acceptance, and behavioral implementation. Specifically, cognitive identification involves stakeholders’ conviction in the validity and efficacy of the best evidence, acknowledging that diarrhea risks arise from modifiable factors rather than inevitable complications. Affective acceptance is demonstrated by healthcare providers’ willingness to undertake diarrhea risk assessment, nutritional status monitoring, and gastrointestinal function documentation even under high-workload conditions, acknowledging that diarrhea prevention benefits patients and is highly necessary. Behavioral implementation refers to stakeholders proactively adhering to bundled interventions (e.g., daily flushing protocols, infusion rate control, and warmed nutritional solutions) while participating in multidisciplinary collaborative decision-making. For instance, medical personnel said:

“Diarrhea is a real problem, and I am very willing to take measures to reduce its occurrence in stroke patients. I hope patients experience no complications during their hospital stay, as this will help them recover faster, which is also beneficial for us.” (Medical staff #4).

##### 3.4.2.4 Exceptional work capabilities

- Strong execution capabilities—refer to healthcare providers’ ability to translate best evidence into concrete actions and integrate best evidence into daily care practices. This capacity emphasizes comprehensive implementation management throughout the entire process, including assessment of diarrhea risk factors, prevention of diarrhea, management of diarrhea, dynamic monitoring, and evaluation of outcomes. For example, medical personnel said:

“The nurses in our department demonstrate exceptional learning capabilities and strong execution skills. They are eager to adopt nursing practices that benefit patient health. I am confident they will perform this project exceptionally well.” (Medical staff #6).

- Strong sense of responsibility—refers to the commitment of neurology medical staff to safeguarding the lives of stroke patients, maintaining a high level of vigilance and rigor in clinical observation, nursing procedures, team collaboration, and health education. For instance, medical personnel said:

“As the head nurse, I believe the strong sense of responsibility among the neurology nurses is a key contributing factor. They demonstrate exceptional dedication to both their work and patients, focusing not only on medical care but also on proactively addressing patients’ emotional needs and mental well-being. Through their own thoughtful approaches, they provide meticulous and comprehensive nursing services.” (Medical staff #4).

- High adapting ability—refers to the ability of medical personnel to respond to changes and flexibly adjust strategies based on clinical experience and the actual condition of stroke patients. Medical personnel said:

“In terms of individual capabilities, I believe medical staff excel at rapid response to emergencies and flexible management of complex conditions, which facilitates the effective implementation of best evidence. When faced with varying scenarios, medical professionals can apply evidence critically and prudently, rather than rigidly applying it without adaptation.” (Medical staff #8).

- Extensive experience in evidence-based practice—refers to healthcare personnel having successfully implemented other best practices at least twice prior to undertaking this project. Medical personnel said:

“We has previously successfully implemented best practices for preventing deep vein thrombosis in stroke patients and preventing pressure ulcers in hospitalized elderly patients, demonstrating extensive experience in translating evidence into clinical practice.” (Medical staff #4).

#### 3.4.3 Third level—practice environment

##### 3.4.3.1 Comprehensive supporting facilities

The neurosurgery department has an adequate supply of enteral feeding pumps and heating devices to provide the necessary resources for preventing diarrhea in stroke patients.

“We have an adequate supply of enteral feeding pumps and heating equipment to ensure immediate availability for each patient when needed.” (Medical staff #2).

##### 3.4.3.2 Non-establishment of a multidisciplinary team

Nutrition, as a key intervention target for restoring neurological function, improving quality of life, and reducing recurrence in stroke patients, is influenced by multiple factors, including psychological state, age, swallowing function, level of consciousness impairment, other comorbidities, and family support. A multidisciplinary collaboration model can integrate perspectives from neurology, nutrition, nursing, rehabilitation, and psychology to implement personalized precision treatment and develop individualized nutritional plans for stroke patients. This approach has a clear role in preventing enteral nutrition complications, improving patients’ neurological function, and enhancing long-term prognosis (20). Medical personnel said:

“Stroke patients present with complex medical conditions, and a single discipline cannot adequately meet their nutritional needs. Currently, we have not established a multidisciplinary team, which is detrimental to the overall management of patients’ nutritional quality.” (Medical staff #6).

##### 3.4.3.3 Insufficient organizational support

- Lack of diarrhea prevention and management systems—indicates that management has not yet established systematic diarrhea prevention and management protocols and workflows. Medical personnel said:

“Everyone has their own operational habits, and without a specific workflow, they might not even realize if a step is missed. We all know that warming infusions helps reduce the incidence of diarrhea, but in practice, the implementation rate of this procedure is not high, which affects the efficiency of healthcare workers.” (Medical staff #5).

- Lack of a quality assessment system for enteral nutrition—refers to the absence of an evaluation system encompassing the three elements of structure, process, and outcome (36). “Structure” refers to the foundational conditions and resource allocation for delivering enteral nutrition; “process” denotes the critical stages and operational protocols for implementing enteral nutrition; while “outcome” signifies the actual effects achieved and the degree of patient benefit derived from enteral nutrition. Medical personnel said:

“Although I recognize the critical importance of nutritional management for patient recovery, certain issues persist in the daily care of enteral nutrition, such as haphazard fixation of nasogastric tubes and the absence of regular assessments of gastrointestinal function. I believe it is necessary to establish systematic quality evaluation metrics to assess the operational quality of each process.” (Medical staff #3).

- Limited educational content and formats for diarrhea

“Sometimes the nurses tell me what to watch out for during enteral nutrition, and I remember it at the time but then forget it later. It would be great if there were printed materials or videos so I could review them repeatedly.” (Stroke patient #2).

##### 3.4.3.4 Interpersonal relationships

- Medical staff collaborate harmoniously

“Doctors and nurses collaborate in a harmonious atmosphere, fostering friendships that make work enjoyable. This creates a positive environment for implementing projects, enabling us to achieve twice the results with half the effort.” (Medical staff #8).

- Support from leader

“This project is highly meaningful, and I fully support it. While it may seem like a minor focus area, if executed properly, it would be incredibly beneficial for everyone. Especially from the patient’s perspective, ensuring adequate nutrition and eliminating the burden of diarrhea significantly enhances their quality of life […]. I have prioritized the incidence of diarrhea in stroke patients as a key focus for quality improvement in nutritional management.” (Medical staff #6).

- Collaboration-oriented interpersonal interaction

“I often ask physical therapists from the Rehabilitation Department to come and provide bedside therapy for stroke patients. We develop rehabilitation plans together, based on each patient’s unique condition. If a patient is feeling low, I set up consultations with psychiatrists. Even though we’re in different departments, I have great working relationships with all my colleagues.” (Medical staff #8).

##### 3.4.3.5 Patients

- Lack of relevant knowledge

“The infusion rate you’ve set for me is far too slow. If I had work this afternoon, I’d increase it myself.” (Stroke patient #2).

“The nurse informed me that once opened, enteral nutrition solutions can only be refrigerated for 24 hours. Any unused portion beyond that time must be discarded. This is difficult for me; I can’t bear to waste it.” (Stroke patient #4).

- Low self-efficacy—which refers to patients’ tendency to doubt their own capabilities, express pessimism about treatment outcomes, distrust medical advice, and resist care measures during diarrhea prevention and management. Patients said:

“When I’m hospitalized, the nurses are there, so I don’t have to worry. But after I’m discharged and go home, it’s just me and my wife. She can’t read, so I take care of her. If I get diarrhea or other problems after going home, what am I supposed to do?” (Stroke patient #3).

“I used to be able to eat on my own, but after the stroke, I suddenly had to rely on a nasogastric tube for nutrition. It was super hard for me to accept this change so fast. I felt annoyed and frustrated, and I just couldn’t listen to what the nurses were saying. I just refused to listen to the nurses when they tried to explain precautions or tell me about my condition.” (Stroke patient #5).

- Diminished abdominal sensitivity—which refers to a reduced ability to perceive sensations within the abdomen, manifested as a lack of sensitivity or delayed response to discomfort. Patients said:

“Before, I could always sense any stomach discomfort right away. Since getting this illness, I feel my digestive system isn’t as strong as before, and my resistance isn’t what it used to be either. Now, even when my stomach feels off, I can’t sense it as quickly.” (Stroke patient #8).

- Cognitive differences—which refer to the fact that each person’s comprehension of the same matter may differ. Correct cognition facilitates disease recovery, while incorrect cognition hinders it. Patients said:

“There’s no need to worry about diarrhea. I used to get it quite often at home and never paid it much mind; it would sort itself out in a couple of days. I’ve never taken any anti-diarrheal medication” (Stroke patient #7).

“I usually like eating cold food, but when I have diarrhea, I become very cautious. I ask my husband to warm up the liquid diet before feeding it to me. I’m afraid that this (diarrhea) will slow down my recovery.” (Stroke patient #4).

- Financial burden

“Everyone says probiotics are good for gut health, but they cost money. For me, I’d definitely prefer to spend as little as possible.” (Stroke patient #3).

- Knowledge sharing among fellow patients—which refers to the process by which patients exchange health information, treatment experiences, or disease management methods to achieve knowledge transfer and mutual support. Patients said:

“Sometimes when I see the nurses are too busy, I feel awkward bothering them. If I have questions, I’ll ask Mr. Li in the next bed; he was a doctor before retiring. He usually gives me great answers to whatever I ask.” (Stroke patient #6).

- Positive rehabilitation mindset—refers to the patient’s inner desire for a swift recovery. This expectation motivates patients to actively cooperate with treatment and enhances their compliance. Patients said:

“When it comes to recovering from illness, I know very little and often don’t know how to take care of myself. Doctors and nurses are the professionals, so I follow everything the nurses tell me. I want to recover quickly so my family won’t have to worry.” (Stroke patient #1).

- Absolute trust in medical professionals

“I remember it clearly. On my first day in the hospital, I felt utterly lost, unsure of what to do, and deeply worried about whether I could recover. It was the nurses’ and doctors’ patient explanations that eased my concerns. That’s why I trust their expertise.” (Stroke patient #8).

### 3.5 Mixed-methods findings

The mixed methods enhanced the integration of survey findings and qualitative research themes, consistent with the OMRU theoretical framework. Additionally, we synthesized a new theme: interpersonal relationships. The mixed-methods findings are shown in Table 6.

**Table 6:** Mixed-methods findings.

Level of OMRU	Survey	Theme	Quantitative	Qualitative	Mixed methods
Evidenced-based innovation					
Development process		/	/	/	//
Innovation attributes		Meeting clinical needs	/	E	/E
	Audit sheet	Some evidence lacks specific implementation standards	B	B	BB
Potential adopters					
Awareness	Audit sheet	Lack of initiative: insufficient awareness of diarrhea risk prevention	B	B	BB
Attitudes		Positive attitudes toward evidence implementation	/	E	/E
Knowledge/skill	Questionnaire	Lack of initiative: knowledge gaps existence	B	B	BB
Concern	Audit sheet	Evidence implementations increase workload	B	B	BB
Current practice	Audit sheet	Exceptional work capabilities	/	E	/E
Practice environment					
Patients	Audit sheet	Patients	(E/B)	(E/B)	(E/B) (E/B)
Cultural/social		/	/	/	//
Structural	Audit sheet	Non-establishment of a multidisciplinary team	B	B	BB
	Audit sheet	Insufficient organizational support	B	B	BB
Economic	Audit sheet	Comprehensive supporting facilities	E	E	EE
Uncontrolled events		/	/	/	//
Additional findings		Interpersonal relationships	/	E	/E

Audit sheet—Supplementary File S1; Questionnaire = knowledge questionnaire on prevention and management of enteral nutrition-related diarrhea in stroke patients (Supplementary File S2); /, not identified; B, barriers; E, enablers.

## 4 Discussion

Prevention of enteral nutrition-related diarrhea should be integrated throughout the entire course of nutritional management for stroke patients. Identifying the gap between clinical practice and best evidence for preventing and managing enteral nutrition-related diarrhea in stroke patients, as well as understanding the barriers and enablers of evidence implementation, may provide a decision-making basis for improving clinical practice and promoting evidence-based nursing. To date, the barriers and enablers to the clinical translation of the best evidence regarding the prevention and management of enteral nutrition-related diarrhea in stroke patients have not been described. This study was conducted to address this gap in knowledge. Guided by the OMRU theoretical framework (27), this study thoroughly examined the barriers and enablers associated with the clinical translation of best evidence for the prevention and management of enteral nutrition-related diarrhea in stroke patients. There are both similarities and differences in clinical practices regarding the prevention and management of enteral nutrition-related diarrhea issues for stroke patients across different levels of healthcare institutions (8, 9, 15). Thus, these barriers and enablers may also be applicable in other nations and areas.

Translating the best evidence for preventing and managing enteral nutrition-related diarrhea in stroke patients into best practice is a complex process. Therefore, it is crucial to identify and address the barriers before implementation. Evidence is the foundation of application, and evidence that has been localized and tailored to the resources of healthcare institutions and actual clinical scenarios can improve the quality of care (17, 37). The interviewees considered that the evidence meets clinical needs, which holds significant clinical importance for improving the quality of EN care for stroke patients. However, at the level of evidence-based innovation, some evidence lacked specific implementation standards, resulting in an inadequate comprehensive assessment of diarrhea risk in stroke patients prior to initiating EN. This failure prevented the early identification of high-risk patients and the implementation of preventive strategies. To enhance the operational feasibility of evidence, we developed a diarrhea risk assessment checklist (see Supplementary File S4) based on the best evidence of the prevention and management of enteral nutrition-related diarrhea in stroke patients (26). At the same time, we uploaded this assessment checklist to the electronic information system to ensure that nurses can conduct a comprehensive assessment of risk factors for diarrhea while effectively reducing the time required for the assessment, thereby translating evidence into clinical practice.

In view of the potential adopters, the primary barriers stemmed from healthcare personnel’s lack of initiative, including insufficient awareness of diarrhea risk prevention and the existence of knowledge gaps. Systematic interventions are needed to improve healthcare providers’ awareness of diarrhea risk prevention. First, specialized training sessions should be organized to reinforce clinical understanding through case studies. Second, risk alerts for enteral nutrition-related diarrhea in stroke patients should be incorporated into electronic order systems and nursing records to raise awareness of prevention. Finally, visual aids such as departmental multimedia equipment and computer desktop backgrounds should be utilized to aid memory and enhance daily implementation. A previous study has pointed out that educational interventions can effectively enhance nurses’ understanding and practice in all aspects of EN, significantly reducing complications and improving the quality of care (38). To enhance healthcare professionals’ knowledge and skills regarding the prevention and management of enteral nutrition-related diarrhea in stroke patients, it was suggested that we should fully leverage existing resources and artificial intelligence to implement diversified, personalized, and targeted educational training programs based on the results of the knowledge survey (39). Concurrently, assessment standards and oversight mechanisms should be established to periodically evaluate training effectiveness (15, 37). In addition, some nurses indicated that procedures such as diarrhea risk assessment, diarrhea-related health education, and monitoring nutritional indicators would increase their workload. Therefore, optimizing diarrhea prevention and management processes, utilizing technological tools to streamline operational procedures, and strengthening team collaboration to reduce the operational burden on nurses are of paramount importance. It was worth noting that healthcare professionals’ positive attitudes to implementing evidence and their exceptional professional competence are the greatest enablers of best practice.

At the practice environment level, comprehensive supporting facilities and positive interpersonal relationships served as key facilitators for optimal evidence translation. Interpersonal relationships were a new and significant influencing factor identified in this study, encompassing harmonious collaboration among medical staff, supportive leadership, and collaboration-oriented interpersonal interaction. Healthcare institutions constitute complex organizations where all medical activities necessitate the involvement of healthcare personnel (40, 41). The successful coordination and collaboration between different services provided by these institutions is directly linked to interpersonal relationships. The more harmonious these relationships, the more readily cooperation succeeds (40). Additionally, translating evidence into clinical practice is an interactive process involving multidisciplinary collaboration (37). Thus, it was suggested that positive interpersonal relationships should be leveraged to establish multidisciplinary teams, with clear delineation of departmental responsibilities and cross-disciplinary resource integration, thereby delivering comprehensive, continuous nutritional therapy and care to stroke patients. In our study, we planned to establish a multidisciplinary team comprising neurologists, specialist neurology nurses, dietitians, rehabilitation therapists, and psychological counselors to jointly manage the nutritional quality of stroke patients. Doolen et al. defined the organizational environment as the management processes, organizational culture, and organizational systems embedded within the parent organization, which can either facilitate or inhibit work performance (42). However, some interviewees in this study pointed out that insufficient organizational support—specifically the lack of diarrhea prevention and management systems and the absence of a quality assessment system for EN—was one of the key barriers to the effective implementation of best evidence. When the organizational environment is unfavorable, a team’s knowledge integration capability is significantly weakened (43). Clear and well-defined workflows are essential for improving the regulation, safety, and efficiency of medical procedures and ensuring that patients receive consistent, high-quality care. Therefore, it is important to establish specific and feasible workflows for the prevention and management of enteral nutrition-related diarrhea in stroke patients based on the best evidence. The institutionalization of EN management is particularly crucial for ensuring its effectiveness and standardization in clinical practice (19, 20). Thus, a quality management indicator system for EN should be established based on the structure-process-outcome model to ensure the safe implementation of EN for stroke patients and to facilitate continuous improvement in nutritional quality.

At the patient level, a lack of relevant knowledge, low self-efficacy, diminished abdominal sensitivity, and financial burden were significant barriers to the implementation of best evidence. The primary beneficiaries of evidence implementation are patients. Thus, patient collaboration and willingness are especially crucial for the advancement of evidence implementation. The effectiveness of health education was influenced not only by the content and formats of diarrhea-related health education but also by differences in patients’ cultural backgrounds, comprehension abilities, and memory capacities (38, 44). Therefore, patient-centered, systematic interventions must be implemented. First, a personalized health education plan for the prevention and management of diarrhea should be developed based on the patient’s age, cultural background, cognitive abilities, memory capacities, and financial situation. Second, multimedia devices are used to present evidence in the form of images and videos to stimulate patients’ interest in learning and enhance learning effectiveness (37). Third, for patients with diminished abdominal sensitivity, the frequency of monitoring objective indicators such as bowel sounds and stool consistency should be increased. In addition, some stroke patients indicated that personal experiences shared by fellow patients in the same ward were more persuasive. Thus, patients with high health literacy who are willing to share information constitute important resources for disseminating evidence. A previous study pointed out that patients with a positive rehabilitation mindset actively sought information, proactively communicated with medical staff, and cooperated fully with nursing care (45, 46). Therefore, efforts should be made to actively identify such patients, train them as peer educators, and promote the dissemination of knowledge regarding the best evidence on the prevention and management of enteral nutrition-related diarrhea in stroke patients.

Managers must recognize that establishing the best evidence of preventing and managing enteral nutrition-related diarrhea in stroke patients as departmental standards requires sustained effort, as altering organizational environments and individual behaviors takes time (15, 47). Thus, we will continue to refine its implementation strategy based on the latest evidence and patients’ evolving needs. In addition, financial burden has been identified as a key barrier, suggesting that policymakers should include products such as human albumin preparations and probiotic supplements in health insurance coverage to reduce the financial burden on patients and improve access to these interventions. More importantly, the analysis of the best evidence and barriers summarized in this study can serve as a reference for updating clinical guidelines on EN care for stroke patients. Health policymakers can incorporate these findings into guideline revisions to establish standard protocols, interventions, and quality control indicators for the prevention and management of diarrhea, thereby standardizing clinical practices and improving the consistency of healthcare services. We have developed other intervention strategies to fully leverage enablers in addition to the aforementioned methods for overcoming barriers (see Supplementary File S5).

## 5 Conclusion

Based on the OMRU theoretical framework, this study systematically examined the barriers and enablers contributing to the gaps between the best evidence and clinical practice regarding the prevention and management of enteral nutrition-related diarrhea in stroke patients. Action strategies to promote the implementation of best evidence should focus on addressing barriers related to the levels of evidence-based innovation, potential adopters, and the practice environment. Implementing these strategies can enhance healthcare providers’ capacity for evidence-based practice, reduce the incidence of enteral nutrition-related diarrhea in stroke patients, and ultimately improve their nutritional status and health outcomes. We must acknowledge the limits of our study. Firstly, due to cultural differences between countries and variations in resources and standards across healthcare institutions of different tiers, baseline audit results may exhibit inconsistencies. Secondly, in the qualitative research, we interviewed only healthcare providers and stroke patients, lacking perspectives from primary caregivers. Thirdly, the study’s sample size was small since data collection was restricted to a single hospital. In the future, multi-center, large-scale studies could be used for validation of the results.

## Data Availability

The original contributions presented in the study are included in the article/Supplementary material, further inquiries can be directed to the corresponding authors.
