# Supplementary material for: Prevention and management of enteral nutrition-related diarrhea in stroke patients: barriers and enablers from best evidence to best practice
Source: Front Nutr. 2026 May 19;13:1816074. doi: 10.3389/fnut.2026.1816074 (PMC13226469; doi:10.3389/fnut.2026.1816074)
Supplement: Supplementary file 1 [file Table_1.DOCX]

Supplementary Material Contents

1. **Supplementary File S1**---Audit sheet for prevention and management of enteral nutrition-related diarrhea in stroke patients
2. **Supplementary File S2**---Knowledge questionnaire on prevention and management of enteral nutrition-related diarrhea in stroke patients
3. **Supplementary File S3**---The interview outline
4. **Supplementary File S4**---Diarrhea risk factors assessment checklist for enteral nutrition in stroke patients
5. **Supplementary File S5**---The implementation intervention strategies of prevention and management of enteral nutrition-related diarrhea in stroke patient

**Supplementary File S1**---Audit sheet for prevention and management of enteral nutrition-related diarrhea in stroke patients.

| **Audit sheet for the prevention and management of enteral nutrition-related diarrhoea in stroke patients** | | | | |
| --- | --- | --- | --- | --- |
| Department： Name： Gender： Age： Admission number： Whether diarrhea occurred during hospitalization：□Yes □No | | | | |
| Marital status： Educational level： Medical payment mode： Family monthly income： Admission date: | | | | |
| Diagnose： Site of lesion： NIHSS score： GCS score： Other diseases： Discharge date： | | | | |
| **Categories** | **Audit criteria** | **Results** | | **Explanation** |
|  |  | Yes | No |  |
| **Organizational management** | 1. The department has formed a multidisciplinary team with clear division of labor to jointly assess the risk of diarrhea during enteral nutrition for stroke patients and provide targeted guidance |  |  |  |
|  | 1. The department has established structure evaluation indicators for enteral nutrition in stroke patients. |  |  |  |
|  | 1. The department has established process evaluation indicators for enteral nutrition in stroke patients. |  |  |  |
|  | 1. The department has established outcome evaluation indicators for enteral nutrition in stroke patients. |  |  |  |
| **Screening for risk factors of diarrhea** | 1. Neurological nurses perform a standardized risk assessment for diarrhea before administering enteral nutrition to stroke patients, evaluating three key aspects: the patient-related factors, the medication-related factors, and the enteral nutrition-related factors.   (1). Patient-related factors: including NIHSS score, GCS score, advanced age, hyperglycemia, hypoalbuminemia, gastrointestinal infection, hemodynamic instability, fasting, Clostridioides difficile infection, lactate deficiency, or fat malabsorption.  (2). Medication-related factors: including antibiotics, proton pump inhibitors, preparations containing sorbitol or electrolytes, acid suppressants, and stool softeners.  (3). Enteral nutrition-related factors: including inappropriate formulations, improper feeding methods, unreasonable infusion concentration/rate/temperature, and microbial contamination. |  |  |  |
| **Multi-dimensional prevention strategy** | 1. For stroke patients with hypoalbuminemia, nurses implement measures to correct hypoalbuminemia in accordance with medical orders. |  |  |  |
|  | 1. Stroke patients with normal serum albumin levels do not require energy or protein supplementation. |  |  |  |
|  | 1. Patients taking probiotics during enteral nutrition.   Probiotic types: |  |  |  |
|  | 1. Nurses maintain the cleanliness of infusion lines and the operating environment during enteral feeding. |  |  |  |
|  | 1. Neurologists select enteral nutrition formulas based on individual patient factors such as gastrointestinal function, comorbidities, and complications.   □Short peptide type □Amino acid pattern □Low sugar □Soluble dietary fiber □Others: |  |  |  |
|  | 1. Once opened, the patient's nutritional preparation is stored at room temperature for less than 4 hours or at 4 °C for less than 24 hours. Any unused portion after 24 hours is discarded. |  |  |  |
|  | 1. Neurologists select enteral feeding routes based on factors including the stroke patient's nutritional risk, swallowing ability, level of consciousness, anticipated duration of enteral nutrition, and risk of complications.   □Oral self-feeding □ Nasogastric tube □ Naso-intestinal tube □ Gastrostomy |  |  |  |
|  | 1. Neurology nurses selected enteral feeding pumps to provide continuous enteral nutrition infusion for patients. The disposable external feeding lines for enteral nutrition are replaced every 24 hours.   □Enteral nutrition pump □ Gravity drip □ Intermittent nasogastric feeding |  |  |  |
|  | 1. Nurses administer enteral nutrition preparations to patients at a temperature of 37-40 °C; for elderly patients, the temperature is maintained between 38-42 °C.   □Ordinary temperature: □Heating temperature : |  |  |  |
|  | 1. The patient's enteral nutrition infusion rate on the first day is 20-50 ml/h; on the second day, the infusion rate is 80-100 ml/h; and within 3-7 days, it is increased to the full target volume.   □Infusion rate: |  |  |  |
|  | 1. The nurse did not add water or coloured substances to the nutritional preparation. |  |  |  |
|  | 1. During enteral feeding, nurses flush the feeding tube with 20-30 ml of warm water every four hours, and prior to and after interrupting feeding or administering medication. |  |  |  |
|  | 1. The neurology department has printed health education materials (including brochures, educational videos, and WeChat public accounts) concerning the prevention and management of enteral nutrition-related diarrhoea in stroke patients. |  |  |  |
|  | 1. During enteral nutrition, nurses provide patients with education on enteral nutrition and assess their knowledge retention through questioning. |  |  |  |
| **Assessment and management of diarrhea** | 1. when a patient's bowel movement frequency exceeds three times per day, nursing staff utilize the Diarrhea Scoring System to assess the patient's diarrhea condition. |  |  |  |
|  | 1. When a patient presents with diarrhea, the nurse assesses abdominal signs, duration of diarrhea, frequency of bowel movements, stool consistency, and perianal skin condition. |  |  |  |
|  | 1. When a patient develops diarrhea, the nurse assesses the patient's electrolyte levels, nutritional status, and mental state. |  |  |  |
|  | 1. When the patient experiences diarrhea, the nurse collects blood and stool samples for laboratory testing in accordance with the doctor's orders. |  |  |  |
|  | 1. When a patient develops diarrhea, the nurse reviews all medications administered to the patient and collaborates with the doctor to make a joint decision regarding the suspension or replacement of the medication causing the diarrhea. |  |  |  |
|  | 1. When the patient experiences diarrhea, the nurse reduces the infusion rate of the enteral nutrition solution. |  |  |  |
|  | 1. When a patient develops diarrhea, the nurse changes the EN formula in accordance with the doctor's orders. |  |  |  |
|  | 1. When the patient experiences diarrhea, the nutritional preparation is stored at a constant temperature of (24.0 ± 1.5) ℃ for over 12 hours before administration. |  |  |  |
|  | 1. When a patient experiences diarrhea, the nurse observes the condition of the perianal skin and applies a skin protectant. |  |  |  |
| **Dynamic monitoring** | 1. When the patient experiences diarrhea, nurses employ the NIHSS score or GCS score to monitor neurological function in stroke patients.   □National Institutes of Health Stroke Scale □Glasgow Coma Scale |  |  |  |
|  | 1. Nurses monitor stroke patients weekly for anthropometric measurements (body mass index, triceps skinfold thickness, muscle condition, etc) and biochemical laboratory parameters (complete blood count, electrolytes, total protein, albumin, prealbumin, etc.).   Anthropometric measurements:  □Muscle condition assessment： (Measure the calf circumference in centimeters)  □BMI： (thinnish：<18.5kg/m^2^ normal: 18.5-24.9kg/m^2^ fat:>25kg/m^2^)  □Triceps Skinfold Thickness,TST： (Use a professional skinfold caliper to locate the midpoint between the acromion and olecranon, then pinch the skin vertically and measure, adult males are usually 8-13mm，adult women are15-25mm)  Biochemical laboratory parameters  □Routine blood test: Hemoglobin (Mild anemia: male 90-120g/L, female 90-110g/L. Moderate anemia: 60-90g/L. Severe anemia: ＜60g/L)  □Total protein: (Normal range：60-80g/L)  □Albumin: (Normal range：adult 40-55g/L，after 60 years old 34-48g/L)  □Prealbumin: (Normal range：adult 280-360mg/L)  □Siderophilin: (Normal range：28.6-51.9μmol/L)  □Retinol binding protein: (Normal range：blood serum 45mg/L, urine 0.04-0.18μmg/L)  □Others: □electrolyte □Plasma amino acid profile □Glycated hemoglobin □C-reactive protein  □immunologic function □vitamin |  |  |  |
|  | 1. Nurses assess and document changes in gastrointestinal function in stroke patients every four hours. |  |  |  |

**Supplementary File S2**---Knowledge questionnaire on prevention and management of enteral nutrition-related diarrhea in stroke patients.

**Knowledge questionnaire on prevention and management of enteral nutrition-related diarrhea in stroke patients---translated version**

Dear Colleagues, I am a postgraduate student at Jiangsu University. We are currently conducting a survey among neurological medical staff to assess their knowledge regarding the prevention of enteral nutrition-related diarrhea in stroke patients. This study aims to understand the current situation and provide evidence for delivering more scientifically sound and comprehensive nutritional support to patients. We kindly request your support in this endeavour. Please note that the survey is anonymous, and your responses will be kept strictly confidential. To ensure the accuracy of this research, we ask that you answer all questions truthfully. Once again, thank you for your support and cooperation! Answering guidelines: Single-choice questions: 4 points each (15 questions total). Correct answers score points; incorrect answers score none. True/False questions: 2 points each (5 questions total). Correct answers score points; incorrect answers score none. Multiple-choice questions: 6 points each (5 questions total). Correct answers score 6 points; incorrect selections score none. Total possible score: 100 points.

**Part One: essential information**

1.Department:

2.Gender:□ male □ female

3.Professional title:

4.Age:

5.Educational background:

6.Years of service:

**Part Two: knowledge questionnaire**

**Single-choice Questions: please select the one (√) that you think is most correct.**

1. Definition of enteral nutrition-related diarrhea：

□A. Watery stools occurred within 48 hours of initiation of enteral nutrition

□B. During enteral nutrition, bowel movements should occur at least three times daily or total volume exceeding 200 g

□C. After confirming the absence of infection, if the volume of stool exceeds 500 ml/day or the frequency of bowel movements increases by ≥2 times/day during nutritional support

□D. Fecal moisture content ＞ 70%, bowel movements 2 times/day

1. When do the guidelines recommend initiating enteral nutrition in stroke patients：

□A. 24～48 h

□B. 36～48 h

□C. 48～72 h

□D. 72～96 h

1. The appropriate infusion temperature for nutritional preparations is：​​

□A. 32 ℃～34 ℃

□B. 34 ℃～36 ℃

□C. 37 ℃～40 ℃

□D.40 ℃～42 ℃

1. The correct initial infusion rate for enteral nutrition on the first day is：

□A .20-50 ml/h

□B. 50-80 ml/h

□C. 60-100 ml/h

□D. 80-100 ml/h

1. The core principle for preventing enteral nutrition-associated diarrhea is：​​

□A. Completely avoid using whole protein formula

□B. Initially, low concentration and low speed infusion was used

□C. Add a broad-spectrum antibiotic

□D. Clean the feeding tube at least 6 times a day

1. What are the correct storage conditions for nutritional preparations

□A. Store at 4 ℃ for 12 h

□B. Store at 4 ℃ for 24h

□C. Store at room temperature for 12h

□D. Store at room temperature for 24h

1. Which disease is a risk factor for diarrhea：​​

□A. Hypertension

□B. Hypoproteinemia

□C. Stable angina

□D. Mild anemia

1. Which types of medication can cause diarrhea：​​

□A. Proton pump inhibitor

□B. Diuretic

□C. Ferrous succinate

□D. Acetaminophen

1. Which component in nutritional preparations is most likely to cause osmotic diarrhea：

□A. Lactin

□B. Medium chain triglycerid

□C. Dietary fiber

□D. Glutamine

1. When patients experience abdominal pain and watery stools during enteral nutrition, the primary measure is：​​

□A. Stop enteral nutrition immediately

□B. Check infusion rate and temperature

□C. Intravenous anisodamine

□D. Collect stool samples for laboratory testing

1. What are the key indicators for assessing the degree of dehydration in patients with diarrhea：

□A. Skin elasticity + urine volume + mental state

□B. Blood pressure + heart rate + respiratory rate

□C. Hemoglobin + Blood Sodium Concentration

□D. Weight loss percentage

1. Regarding the relationship between enteral nutritional diarrhea and antibiotics, the correct statement is：​​

□A. All antibiotics caused enteric nutritional diarrhea

□B. Broad-spectrum antibiotics disrupt gut flora and increase risk

□C. The use of antibiotics intravenously does not affect enteral nutrition

□D. Metronidazole can completely prevent enteric nutritional diarrhea

1. For patients with enteral nutrition-related diarrhea, which aspects should be prioritized for monitoring：​​

□A. Serum prealbumin + electrolytes

□B. Cardiomyosin + Electrolytes

□C. Trace elements and electrolytes

□D. Coagulation + Electrolytes

1. The normal range for albumin in adults is：

□A. 30-45 g/L

□B. 40-55 g/L

□C. 45-55 g/L

□D. 50-60 g/L

15. Which intervention is ineffective for enteral nutrition-related diarrhea：​​

□A. Switch to short peptide formula

□B. Add probiotics

□C. Infusion rate reduced by half

□D. Switch to whole protein formula

**True-False Questions: please write your answer in the brackets.**

16. Patients with diarrhea should immediately take anti-diarrheal medication.（ ）

17. The severity of stroke patients' condition is unrelated to diarrhea.（ ）

18. Serum prealbumin is a reliable indicator for assessing long-term nutritional status.（ ）

19. Routine supplementation with energy or protein preparations does not increase the risk of diarrhea in stroke patients with normal albumin levels.（ ）

20. Water or medication may be added to nutritional preparations.（ ）

**Multiple-choice Questions: please select the one or more (√) that you think is most correct.**

1. What are the common causes of enteral nutrition-related diarrhea：

□A.The osmotic pressure of the nutrient solution is too high

□B. The patient is lactose intolerant

□C. Infusion rate is too fast

□D. The patient is currently hemodynamically unstable

□E. The infusion temperature is appropriate

1. Key measures for preventing enteral nutrition-related diarrhea：

□A. Start infusion at low concentration and slow speed

□B. Add antibiotics routinely

□C. Completely avoid fiber formulas

□D. Use only parenteral nutrition

□E. Heat infusion

1. Which nutritional preparation is suitable for patients with indigestion：

□A. Short peptides

□B. Whole protein

□C. High unsaturated fatty acids

□D. Glutamine

□E. amino acid

1. What measures can be taken to improve enteral nutrition-related diarrhea：

□A. Switch to isotonic/low-osmotic formula

□B. Switch to lactose-containing formula

□C. Add probiotics

□D. Reduce the infusion volume to 20-50 ml/h

□E. Increase total infusion volume

1. What are the nutritional monitoring indicators for stroke patients

□A. Albumin

□B. Prealbumin

□C. Total protein

□D. Transferrin

□E. Retinol binding protein

**Supplementary File S3**---The interview outline

| **The interview outline** | |
| --- | --- |
| **Medical staff** | **Stroke patients** |
| 1. Do you think it is necessary to implement enteral nutrition-related diarrhea prevention and management for stroke patients? Why? 2. How do you think the applicability of using the best evidence for the prevention and management of enteral nutrition-related diarrhea in stroke patients in your institution? Why? 3. How do you think the feasibility of using the best evidence for the prevention and management of enteral nutrition-related diarrhea in stroke patients? Why? 4. What factors at the individual or departmental level currently hinder your implementation of this evidence in your institution? 5. Can you suggest some strategies or approaches to overcome the above barriers? 6. What further training do you think necessary to implement this project? 7. What factors at the individual or departmental level currently help your implementation of this evidence in your institution? 8. What else do you think needs to be changed to implement this program? 9. What else do you want to supplement in this topic? | 1. What do you know about the prevention and management of enteral nutrition-related diarrhea? 2. How important do you think preventing enteral nutrition-related diarrhea is? Why? 3. Has your healthcare provider talked to you about preventing enteral nutrition-related diarrhea? When? How? 4. What measures have been taken to prevent enteral nutrition-related diarrhea during your hospitalization? 5. Do you think the current education on the prevention of diarrhea is helpful? Why? 6. What difficulties did you encounter in receiving guidance on the prevention of enteral nutrition-related diarrhea? 7. What factors can help you adopt our recommendations for prevention and management of enteral nutrition-related diarrhea? 8. What else would you like to know about enteral nutrition? |

**Supplementary File S4**---Diarrhea risk factors assessment checklist for enteral nutrition in stroke patients

| **Diarrhea risk factors assessment checklist for enteral nutrition in stroke patients** | | |
| --- | --- | --- |
| Department： Name： Gender： Age： Admission number： | | |
| Temperature: Pulse: Breathe: Heart rate: | | |
| **Risk assessment** | **Before enteral nutrition infusion** | |
|  | **☞Patient-related factors：**  □Stroke severity(NHISS ＞ 15 or GCS ≤ 8)  □Age ≥ 65 years  □Hyperglycemia  □Hypoalbuminemia  □Gastrointestinal infection  □Hemodynamic instability  □Fasting  □Clostridioides difficile infection  □Lactate deficiency  □Fat malabsorption  **☞Medication-related factors：**  □Antibiotics  □Proton pump inhibitors  □Preparations containing sorbitol  □Preparations containing electrolytes  □Acid suppressants  □Stool softeners  □Prokinetic drugs  □Sedatives  □Oral potassium preparations  **☞Enteral nutrition-related factors：** | □Inappropriate formulations: enteral nutrition preparation fat content ＞ 20 %  □Improper feeding methods: intermittent nasogastric feeding  □unreasonable infusion concentration: enteral nutrition osmotic pressure ＞ 400 mOsm/L or enteral nutrition preparation fat content ＞ 20 %  □unreasonable infusion rate: ＞ 100 mL/h  □unreasonable infusion temperature: □ ≤ 36℃ □ ≥ 42℃  □microbial contamination  **☞Other risks**  □Oral energy or protein supplements  □Add water or coloured substances to the nutritional preparation  □Maintain the cleanliness of infusion lines and the operating environment  □Flush the feeding tube with 20-30 mL of warm water before starting enteral nutrition  □Correct nutrition preparation storage temperature  □Emphasize the precautions of enteral nutrition infusion to patients  □Nutritional preparation daily infusion volume ≥ 1000 ml  ***Notes:*** If applicable, mark the corresponding box with a "√". |

**Supplementary File S5**---The implementation intervention strategies of prevention and management of enteral nutrition-related diarrhea in stroke patients

| **The implementation intervention strategies of prevention and management of enteral nutrition-related diarrhea in stroke patients** | | |
| --- | --- | --- |
| **Levels** | **Strategies** | **Measures** |
| Barrier management | 1 Build a multidisciplinary team | - Establish a multidisciplinary team to manage the nutritional care of stroke patients. Team members include neurologists, specialist neurological nurses, dietitians, rehabilitation therapists, and psychological counselors. The responsibilities of members of the multidisciplinary team are clearly defined. |
|  | 2 Formulate specific implementation standards | - Establishment of enteral nutrition structure evaluation indicators:  1. Formulate enteral nutrition management systems, protocols and procedures, clarifying operational standards. 2. Develop training and assessment criteria, specifying training content and assessment focal points. 3. Provide sufficient enteral feeding pumps. 4. Establish evaluation metrics: including the implementation rate of regular training by the nutrition team, the pass rate for enteral nutrition knowledge assessments among nursing staff, and the ratio of enteral feeding pumps to feeding sets.  - Establish evaluation indicators for enteral nutrition processes:  1. Develop standards and procedures for nutritional risk screening, nutritional status assessment, dysphagia screening and evaluation, and aspiration risk assessment; 2. Standardize the placement and maintenance of enteral feeding tubes, ensuring correct implementation of enteral feeding; 3. Develop evaluation metrics: including implementation rates for nutritional risk screening, nutritional status assessment, aspiration risk assessment, and dysphagia assessment; compliance rates for elevating the head of the bed ≥30°during feeding; execution rates for confirming feeding tube placement; accuracy rates for infusion rates; correctness rates for tube fixation; and correctness rates for tube flushing procedures.  - Establishment of enteral nutrition outcome evaluation indicators:  1. Develop a review system and procedures, clearly define monitoring content, analyse and summarize findings based on outcomes, and continuously improve quality; 2. Formulate evaluation indicators: including aspiration incidence rate, diarrhoea incidence rate, constipation incidence rate, unplanned feeding tube removal rate, and feeding tube occlusion rate.use, so as to be used and learned by medical staff. The electronic version of the manual is distributed to all nurses in the department, and the paper version is printed and bound and placed in the department for nursing staff to read and learn at any time. |
|  | 3 Carry out training and examination to medical staff | - Design and implement training and assessment programmes for healthcare personnel, covering knowledge and skills pertaining to the prevention and management of enteral nutrition-related diarrhea, with regular training sessions and assessments organized. |
|  | 4 Use diarrhea assessment checklist for risk factor screening | - In terms of prevention, risk identification should be carried out with consideration of four factors:  1. Patient factors 2. Drug factors 3. enteral nutrition-related factors 4. Others  - A structured assessment tool should be used to screen the risk factors of diarrhea in stroke patients, classify them into high, medium and low risk, and hang signs. Personalized prevention strategies were formulated according to the assessment results. For example, adjusting the infusion rate and temperature to an appropriate level. - Integrate the diarrhea risk classification system into the hospital information system, display it on the bedside screen, and input the audit sheet into the information system to improve work efficiency. |
|  | 5 Optimize the health education scheme | - Patients and their families are invited to participate in the development of educational materials and update relevant knowledge regularly. - Create an illustrated diarrhea prevention education manual, adapt it into a patient-favorite song, and include a QR code for scanning to play the education video or audio. - The content of the propaganda is synchronized with the department's visual equipment and played regularly. - Increase the frequency of education, including admission education, before and after daily enteral nutrition operation, discharge and follow-up. - Identify knowledge acquisition channels, such as promotional brochures, official accounts, WeChat groups, face-to-face consultations, and post-discharge follow-up calls. - establishing WeChat groups for regular dissemination of high-quality enteral nutrition content, with designated patients sharing insights on each post to foster two-way interaction; - Select appropriate education methods according to patient preferences, such as paper manuals, videos, audio, and knowledge contests among patients. - The feedback method was used to assess the degree of knowledge mastery of patients and their families. - Actively communicate with patients, ask their opinions and suggestions, and improve the acceptance of evidence from the perspective of patients. |
|  | 6 Develop a handbook on enteral nutrition-related diarrhea prevention | - The handbook should include evidence content, procedures, tools and instructions for use, so as to be used and learned by medical staff and stroke patients. The electronic version of the manual is distributed to all medical staff members in the department. Place the diarrhea prevention education manual on the corridor and ward door education boards, and play the education videos on the department's visual equipment. When patients are discharged, distribute personalized education materials again. - Monthly enteral nutrition knowledge salons are held, with awards established to encourage active participation from medical staff, healthcare providers and patients. |
|  | 7 Increase the frequency of patrols | - For patients with diminished abdominal sensitivity, healthcare personnel should increase the frequency of rounds to enable early detection of abnormalities. |
|  | 8 Peer support among fellow patients | - Encourage patients to support one another and learn collectively, organising regular discussion sessions to share experiences. - Placing patients with negative emotions alongside optimistic patients in the same ward enhances confidence in recovery and self-efficacy. |
| Transfer | 9 Utilize information technology to facilitate the conversion of evidence | - Utilize information technology to develop an elderly-friendly enteral nutrition tracking mini-program. Its features include knowledge links, enteral nutrition records, self-assessment of nutritional status, and one-click consultation. - Provide one-to-one explanations of enteral nutrition-related knowledge to patients. - During hospitalization, teach patients how to use the mini-program. |
|  | 10 Alleviate patients' financial burden | - Use of medicines covered by medical insurance and respect for patient preferences. |
| Follow-up | 11 follow-up | - For patients without smartphones, establish an information booklet and conduct regular telephone follow-ups to monitor their enteral nutrition status. - By an elderly-friendly enteral nutrition tracking mini-program, monthly backend statistics evaluate post-discharge enteral nutrition quality. For patients with poor compliance, we conduct phone follow-ups and provide personalized guidance based on their needs. |
